# Genome of *Cupriavidus* sp. HMR-1, a Heavy Metal-Resistant Bacterium

**DOI:** 10.1128/genomeA.00202-12

**Published:** 2013-02-14

**Authors:** Li-Guan Li, Lin Cai, Tong Zhang

**Affiliations:** Environmental Biotechnology Laboratory, Department of Civil Engineering, University of Hong Kong, Hong Kong SAR, China

## Abstract

*Cupriavidus* sp. HMR-1 was isolated from a heavy metal-enriched culture of activated sludge from a wastewater treatment plant in Hong Kong. Here, we release the HMR-1 genome to provide basic genetic characteristics for a better understanding of its multiple heavy metal resistance properties.

## GENOME ANNOUNCEMENT

Organisms of the genus *Cupriavidus* (formerly named *Wautersia*, *Ralstonia*, or *Alcaligenes*) are Gram negative, have peritrichously flagellated rods, and are chemoheterotrophic or chemolithotrophic (1). Much attention has been paid to *Cupriavidus* isolates due to their capabilities to biodegrade recalcitrant compounds and xenobiotics (2–5). For instance, *Cupriavidus eutropha* JMP134 can grow well on chlorinated aromatic pollutants, e.g., 2,4,6-trichlorophenol (4). In addition to their biodegradation capabilities, several *Cupriavidus* isolates have been identified further as multiple-heavy metal-resistant bacteria, such as *Cupriavidus metallidurans* CH34 and *Cupriavidus necator* N-1 (6, 7). A commonly found feature in *Cupriavidus* is the existence of large plasmids, where mobile genetic elements, biodegradation genes, and multiple heavy metal resistance genes are always located (8, 9). *C. metallidurans* CH34 was isolated as a cadmium-resistant bacterium in 1976 in Belgium from the sludge of a decantation tank with high concentrations of several heavy metals. Its plasmids, pMOL28 (171,459 bp), carrying genes that are responsible for resistance to Co(II), Cr(VI), Hg(II), and Ni(II), and pMOL30 (233,720 bp), carrying genes that are involved in resistance to Ag(I), Cd(II), Co(II), Cu(II), Hg(II), Pb(II), and Zn(II), have been well studied (8). Here, *Cupriavidus* sp. HMR-1 was isolated from a heavy metal-enriched culture of activated sludge from a wastewater treatment plant in Hong Kong. We believe that the sequencing of the *Cupriavidus* sp. HMR-1 genome is helpful in understanding the evolution of such bacteria and in exploring the potential mechanisms for adaptation to environmental pressures.

Whole-genome shotgun sequencing of *Cupriavidus* sp. HMR-1 was performed by the Beijing Genomics Institute (BGI) using the Illumina HiSeq 2000 platform (Illumina, Inc.). A total of 3.5 Gbp of clean data was obtained from BGI after filtering low-quality reads. The quality of reads was further examined with the FastQC program (v0.10.1). After a quality check, the *de novo* genome assembly of short reads was carried out using the CLC Genomics workbench v4.9 using a word size of 26 bp; this yielded 287 contigs that were >1,000 bp (*N*_50_, 46,896 bp). The genome of *Cupriavidus* sp. HMR-1 was evaluated to be 6,644,483 bp, which was sequenced with >500-fold genome coverage. HMR-1 genome annotation was performed by the NCBI Prokaryotic Genomes Automatic Annotation Pipeline (PGAAP). The results revealed that the HMR-1 draft genome has 6,231 coding sequences (CDSs), including 6,174 protein-coding genes, 53 tRNA genes, and 4 rRNA genes, with 63% G+C content.

### Nucleotide sequence accession numbers.

This Whole Genome Shotgun project has been deposited at DDBJ/EMBL/GenBank under the accession no. ANKP00000000. The version described in this article is the first version, ANKP01000000.
